# The mediating role of inflammation-related indicators in the association of remnant cholesterol with gestational diabetes mellitus

**DOI:** 10.7189/jogh.15.04172

**Published:** 2025-06-20

**Authors:** Lihua Lin, Juan Lin, Jianying Yan, Xiaomei Wang

**Affiliations:** 1 Department of Healthcare, Fujian Maternity and Child Health Hospital, College of Clinical Medicine for Obstetrics, Gynaecology and Paediatrics, Fujian Medical University, Fuzhou, China; 2 Department of Obstetrics, Fujian Maternity and Child Health Hospital, College of Clinical Medicine for Obstetrics, Gynaecology and Paediatrics, Fujian Medical University, Fuzhou, China

## Abstract

**Background:**

Gestational diabetes mellitus (GDM) is a common complication during pregnancy, posing threats to both maternal and infant health. We aimed to investigate the association of remnant cholesterol (RC) and GDM during the first trimester of pregnancy through a prospective cohort study, and to explore the mediating effects of inflammation-related indicators.

**Methods:**

We analysed data including 13 446 pregnant women and employed a generalised linear model to estimate the relative risk (RR) and 95% confidence intervals (CI) for the associations between RC level and GDM. We used the restricted cubic spline analyses to reflect the dose-response relationship. We made mediation analyses to explore the mediating effects of inflammation-related indicators on the relationship between RC level and risk of GDM.

**Results:**

The overall incidence of GDM was 22.3%, and this incidence increased across the RC quartiles, reaching 25.7% for the highest quartile of RC levels. There was a linear upward trend in the risk of GDM with increasing RC levels during the first trimester of pregnancy (*P* < 0.001 and for nonlinearity *P* = 0.124). Compared to the lowest RC quartile, higher RC quartiles were linked with an increased risk of GDM, with quartile two RR = 1.20 (95% CI = 1.07–1.36), quartile three RR = 1.24 (95% CI = 1.11–1.40), and quartile four RR = 1.48 (95% CI = 1.32–1.67), in the crude model. This positive association persisted even when total cholesterol, triglycerides, low-density lipoprotein cholesterol, and high-density lipoprotein cholesterol levels were within normal ranges, and it was consistent across groups stratified by maternal advanced age, occupation, gravidity, parity, pre-pregnancy body mass index, and hypertensive disorder of pregnancy. Moreover, inflammation-related indicators, including leukocytes and neutrophils, partially mediated these associations, accounting for 4.89% and 6.60% of the mediation proportion, respectively.

**Conclusions:**

Higher serum RC levels in early pregnancy were positively associated with an increased risk of developing GDM. Leukocytes and neutrophils partially mediated these associations. RC may serve as an early predictor of GDM, and monitoring RC may help optimise GDM prevention.

Gestational diabetes mellitus (GDM) is one of the most common complications during pregnancy, posing short- or long-term threats to both maternal and infant health. Pregnant women with GDM are at a higher risk of developing type two diabetes and several other metabolic disorders later in life, all of which are risk factors for cardiovascular disease [1–4]. Additionally, children born to mothers with poorly managed GDM are at an increased risk of developing obesity, diabetes, and several other cardiovascular risks in adolescence and throughout adulthood [5]. Worldwide, around 14% of pregnant women are affected by GDM, with approximately 15% of women suffering from GDM in mainland China [6,7]. Accumulated findings have shown that risk factors for GDM include maternal overweight or obesity, excessive gestational weight gain, old maternal age, a history of GDM and a family history of diabetes [8]. The direct costs associated with GDM complications, such as neonatal metabolic and maternal and hypertensive disorders, are reported to be USD 1.6 billion [6]. Screening for and treating GDM can significantly impact costs, particularly in terms of the costs of maternal and neonatal morbidities. Several studies have indicated that incorporating screening and intervention for GDM can reduce the chances of perinatal complications and potentially prevent long-term health issues [1]. Therefore, it is crucial to consistently identify modifiable risk factors for GDM. Lipids play a crucial role in the development of GDM [9–11]. Previous studies have primarily focused on traditional lipid parameters such as low-density lipoprotein cholesterol (LDL-C), high-density lipoprotein cholesterol (HDL-C), and triglyceride (TG) in relation to pregnancy-related complications. Recently, remnant cholesterol (RC) has emerged as a useful marker for recognising diabetes mellitus and other cardiovascular diseases [12,13]. Relevant research has shown that the role of RC beyond the LDL-C in the contribution to DM, with females being more sensitive to DM from RC than males [14]. RC comprises cholesterol that remains in the blood after the body has utilised it in various physiological processes, including very low-density lipoproteins, intermediate-density lipoproteins, and chylomicron remnants [15,16]. RC contains more cholesterol, which is thought to be more harmful to pancreatic beta cells and is more closely associated with insulin resistance [17,18]. Some studies suggest that non-HDL-C is more effective than traditional cholesterol markers such as LDL-C in predicting diabetes risk [19]. Additionally, elevated RC promotes higher levels of inflammation, which subsequently leads to insulin resistance [17,20,21].

To date, limited research has explored the relationship between RC and risk of GDM, and further validation of these associations in China, especially in Southeast China, is warranted [22,23]. Besides, methodological limitations in existing studies, such as small sample sizes and a lack of dose-response analysis, need to be addressed. Based on these gaps in the literature, we hypothesised that RC levels during the first trimester of pregnancy may be linked with GDM risk. We aimed to explore the associations between RC levels in the first trimester of pregnancy and the risk of GDM through a prospective cohort study.

## METHODS

### Study population

We conducted the analysis based on data from a prospective birth cohort study in Fujian province, China. This birth cohort study aimed to investigate the impact of the environment, lifestyle, and nutrition on the health of pregnant women and their offspring. Pregnant women who had their first antenatal care visit before 13 weeks of gestation and planned to deliver at Fujian Maternity and Child Health Hospital were recruited and regularly followed up by trained investigators. The Fujian Maternity and Child Health Hospital ethics committee approved the birth cohort study (approval number 2017-030-02). Written informed consent was obtained from all participants upon enrolment.

**Figure 1:**
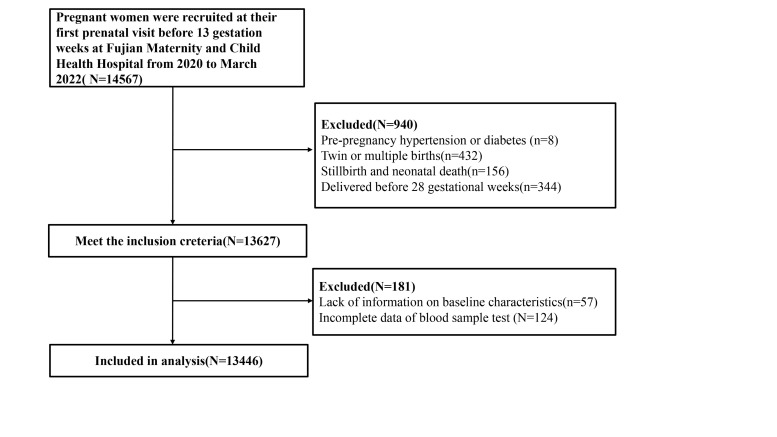
The inclusion and exclusion criteria of the study population.

The data used for this analysis met the following additional criteria: 1) singleton pregnancies, 2) complete data on essential baseline information, oral glucose tolerance test outcomes, lipid results during the first trimester, and 3) absence of pre-existing diabetes, previous GDM, severe cardiovascular, liver, kidney, or autoimmune disease. Between 2020–22, 14 567 pregnant women had their first prenatal visit before 13 weeks of gestation, completed all prenatal visits, and delivered at Fujian Maternity and Child Health Hospital. Finally, 13 446 pregnant women met the inclusion criteria for analysis (**Figure 1**).

### Lipid measurements and definition

We collected blood samples after an overnight fast, and we tested total cholesterol (TC), TG, HDL-C, and LDL-C levels in the biochemistry laboratory of the Fujian Maternity and Child Health Hospital. We calculated the RC level (mmol/L) as TC (mmol/L) minus LDL-C (mmol/L) minus HDL-C (mmol/L) [24].

### Ascertainment of GDM

Oral glucose tolerance test was conducted at 24–28 weeks of gestation, and GDM was diagnosed based on the following criteria: a fasting plasma glucose level ≥2.1 mmol/L, a one-hour plasma glucose level ≥10.0 mmol/L, or a two-hour plasma glucose level ≥8.5 mmol/L [25].

### Covariates

Referring to the selection of covariates in the relevant literature and considering the possible confounders of GDM, we selected the following variables as covariates: maternal age, educational level, occupation, parity, gravidity, pre-pregnancy body mass index (BMI), and hypertensive disorders of pregnancy (HDP) (yes or no) [26–28]. Pre-pregnancy weight was reported by the pregnant women at enrolment. We calculated pre-pregnancy BMI as pre-pregnancy weight (in kg) divided by the square of height (in m^2^) and we categorised it as underweight (BMI < 18.5 kg/m^2^), normal-weight (18.5 ≤ BMI < 24 kg/m^2^), and overweight or obese (BMI ≥ 24 kg/m^2^) based on the Chinese adult BMI classification [29]. Total gestational weight gain was the difference between the maternal weight at delivery (kg) and the pre-pregnancy weight (kg).

### Statistical analysis

We stratified the study population based on levels of RC in quartiles (Q), each containing approximately one-fourth of the study population: the lowest quartile (Q1), the 25–50 percentile quartile (Q2), the 50–75 percentile quartile (Q3), and the highest quartile (Q4). We presented baseline characteristics of participants as means (x̄) and standard deviations (SD) for continuous variables and numbers (percentages) for categorical variables. To compare baseline characteristics among the RC levels quartiles, we used analysis of variance for continuous variables with a normal distribution or the Kruskal-Wallis test for skewed distribution, and χ^2^ tests for categorical variables. We calculated the generalised linear models to examine the relative risks (RRs) and 95% confidence intervals (CIs) across the baseline RC level and the risk of GDM. We adjusted covariates, and constructed two models. Model 1 was the crude model, while Model 2 was adjusted for maternal age (continuous variable), educational level (college and above, high school or equivalent, and less than high school), occupation (yes or no), parity (primiparity or multiparity), gravidity (one, two, and ≥3), pre-pregnancy BMI, and HDP (yes or no). Additionally, we further investigated the association between RC level and the risk of GDM with normal levels of TC, LDL-C, and HDL-C.

We developed a restricted cubic spline regression model to explore the dose-response relationship and the linear or nonlinear shapes of the relationship between RC level and the risk of GDM. We conducted subgroup analyses based on maternal age (<35 and ≥35 years), occupation (yes or no), gravidity (one, two, and ≥3), parity (primiparity or multiparity), pre-pregnancy BMI (underweight, normal weight, overweight, or obese), and HDP (yes or no) to evaluate the differential effects of RC level across various baseline characteristics. Further, we performed stratified analysis based on pre-pregnancy BMI categories. We conducted mediation analyses using a SAS macro (SAS Institute, Cary, North Carolina, USA) package ‘%mediation’ to explore the potential mediating effects of inflammation-related indicators (leukocytes, neutrophils, lymphocytes, and monocytes) on the relationship between RC level and the risk of GDM. The average direct effect referred to the effect of baseline RC level on GDM risk without a mediator, while the average indirect effect referred to the effect of baseline RC level on GDM risk through a mediator. We calculated the proportion of mediation by dividing the indirect effect by the total effect. If both the total effect and indirect effect were significant and the proportion of mediation was positive, it suggested that a mediating effect exists [30,31].

Moreover, we conducted several sensitivity analyses to assess the robustness of the results. First, we stratified the RC levels based on tertiles. Second, considering the significant association between gestational weight gain and GDM, we excluded individuals from the study population who had excessive gestational weight gain before the diagnosis of GDM. We considered a two-tailed *P*-value <0.05 statistically significant.

## RESULTS

### Characteristics of the study population

The average maternal age was x̄ = 30.4 years (SD = 4.4), height was x̄ = 159.9 cm (SD = 5.7), and pre-pregnancy weight was x̄ = 53.3 kg (SD = 7.1). The proportions of employment, gestational weight gain categories, caesarean section, parity, gravidity, and GDM were significantly different among the RC quartile groups (**Table 1**). Moreover, the highest RC level had a higher proportion of multiparity, and higher gravidity (*P* < 0.05). Among the total study population, 2994 pregnant women developed GDM, and the overall incidence of GDM was 22.3%. The incidence of GDM increased across the RC quartiles, with 18.9% in Q1, 21.7% in Q2, 22.6% in Q3, and 25.7% in Q4, indicating a higher incidence of GDM in the higher RC levels.

**Table 1:** Characteristics of the study population according to quartiles of RC*

Variables	Total (n = 13 446)	Q1 (n = 3287)	Q2 (n = 3381)	Q3 (n = 3358)	Q4 (n = 3420)	*P-*value
Maternal age, x̄ (SD)	30.4 (4.4)	30.4 (5.1)	30.3 (4.6)	30.3 (4.0)	30.6 (4.0)	0.023
Occupation						<0.001
*Yes*	4409 (32.8)	1164 (35.4)	1125 (33.3)	1073 (32.0)	1047 (30.6)	
*No*	9037 (67.2)	2123 (64.6)	2256 (66.7)	2285 (68.0)	2373 (69.4)	
Maternal height in cm, x̄ (SD)	159.9 (5.7)	159.8 (5.7)	159.8 (5.5)	159.9 (5.6)	160.0 (5.8)	0.44
Pre-pregnancy weight in kg, x̄ (SD)	53.3 (7.1)	53.3 (7.2)	53.1 (7.0)	53.2 (7.0)	53.6 (7.3)	0.007
BMI group†						0.505
*Underweight*	2333 (17.4)	578 (17.6)	595 (17.6)	599 (17.8)	561 (16.4)	
*Normal weight*	9596 (71.4)	2333 (71.0)	2421 (71.6)	2393 (71.3)	2449 (71.6)	
*Overweight and obese*	1517 (11.3)	376 (11.4)	365 (10.8)	366 (10.9)	410 (12.0)	
Gestational weight gain						0.044
*As recommended*	3309 (24.6)	770 (23.4)	806 (23.8)	818 (24.4)	915 (26.8)	
*Below recommended*	3435 (25.5)	845 (25.7)	864 (25.6)	878 (26.1)	848 (24.8)	
*Above recommend*	6702 (49.8)	1672 (50.9)	1711 (50.6)	1662 (49.5)	1657 (48.5)	
Caesarean section						<0.001
*No*	8961 (66.6)	2066 (62.9)	2263 (66.9)	2295 (68.3)	2337 (68.3)	
*Yes*	4485 (33.4)	1221 (37.1)	1118 (33.1)	1063 (31.7)	1083 (31.7)	
Parity						<0.001
*Primiparity*	8803 (65.5)	2353 (71.6)	2314 (68.4)	2123 (63.2)	2013 (58.9)	
*Multiparity*	4643 (34.5)	934 (28.4)	1067 (31.6)	1235 (36.8)	1407 (41.1)	
Gravidity						<0.001
*1*	6302 (46.9)	1555 (47.3)	1712 (50.6)	1569 (46.7)	1466 (42.9)	
*2*	3929 (29.2)	890 (27.1)	911 (26.9)	1026 (30.6)	1102 (32.2)	
*≥3*	3215 (23.9)	842 (25.6)	758 (22.4)	763 (22.7)	852 (24.9)	
GDM						<0.001
*No*	10 452 (77.7)	2666 (81.1)	2621 (77.5)	2623 (78.1)	2542 (74.3)	
*Yes*	2994 (22.3)	621 (18.9)	735 (21.7)	760 (22.6)	878 (25.7)	
HDP						0.189
*No*	12 585 (93.6)	3067 (93.3)	3151 (93.2)	3169 (94.4)	3198 (93.5)	
*Yes*	861 (6.4)	220 (6.7)	230 (6.8)	189 (5.6)	222 (6.5)	

BMI – body mass index, GDM – gestational diabetes mellitus, HDP – hypertensive disorders of pregnancy, RC – remnant cholesterol, SD – standard deviation, Q – quartile, x̄ – mean

*Presented as n (%) unless specified otherwise.

†Underweight (BMI < 18.5 kg/m^2^), normal-weight (18.5 ≤ BMI < 24 kg/m^2^), and overweight or obese (BMI ≥ 24 kg/m^2^).

### Association between RC level and risk of GDM

**Figure 2:**
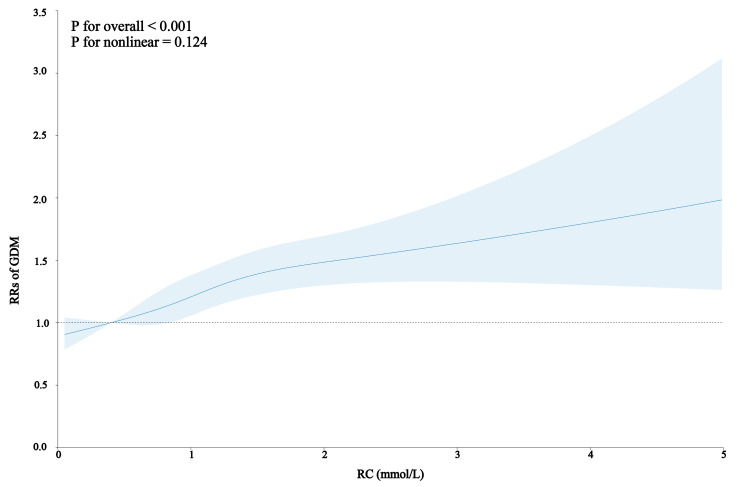
Dose-response relationship between RC level in the first trimester and risk of GDM. The solid line and shadow part represent the unadjusted probability and adjusted 95% CIs. Adjusted for maternal age, educational level, occupation, parity, gravidity, pre-pregnancy BMI, and hypertensive disorders of pregnancy. GDM – gestational diabetes mellitus, RC – remnant cholesterol, RR – relative risk.

An increasing trend of GDM was observed with increasing RC levels, both with and without adjustment for confounding factors (overall *P* < 0.001 and for nonlinearity *P* = 0.124) (**Figure 2**). We performed a generalised linear model to explore the association between RC levels and the risk of GDM, while adjusting for potential confounding factors. Initially, when comparing pregnant women in the lowest RC level (Q1) with those in Q2, Q3, and Q4, the RRs for the risk of GDM were RR = 1.20 (95% CI = 1.07–1.36), RR = 1.24 (95% CI = 1.11–1.40), and RR = 1.48 (95% CI = 1.32–1.67), respectively, in the crude model. After adjusting for sociodemographic characteristics, the trends of RRs remained consistent. Additionally, in the adjusted models, the risk of GDM was RR = 1.24 (95% CI = 1.15–1.33) for each unit increase in RC level. Similarly, the risk of GDM was RR = 1.13 (95% CI = 1.08–1.17) for each SD increase in RC level (**Table 2**).

**Table 2:** Associations between maternal RC levels in the first trimester and the risk of GDM

		Model 1*		Model 2†	
**Variable**	**GDM, n (%)**	**RR (95% CI)**	***P-*value**	**RR (95% CI)**	***P-*value**
RC per unit	2994 (22.3)	1.23 (1.15–1.32)	<0.001	1.24 (1.15–1.33)	<0.001
RC per SD	2994 (22.3)	1.12 (1.08–1.17)	<0.001	1.13 (1.08–1.17)	<0.001
RC quartile					
*Q1*	621 (18.9)	ref		ref	
*Q2*	735 (21.7)	1.20(1.07–1.36)	<0.001	1.25 (1.11–1.42)	<0.001
*Q3*	760 (22.6)	1.24 (1.11–1.4)	0.002	1.28 (1.14–1.45)	<0.001
*Q4*	878 (25.7)	1.48 (1.32–1.67)	<0.001	1.52 (1.35–1.71)	<0.001
*P*-value for trend			<0.001		<0.001

CI – confidence intervals, GDM – gestational diabetes mellitus, RC – remnant cholesterol, ref – reference, RR – relative risk, Q – quartile

*Model 1 crude.

†Model 2 adjusted for maternal age, educational level, occupation, parity, gravidity, pre-pregnancy BMI, and hypertensive disorders of pregnancy.

### Association between RC level and risk of GDM with normal TC, TG, LDL-C, HDL-C levels

The RRs were RR = 1.18 (95% CI = 1.07–1.31) in the crude model and RR = 1.22 (95% CI = 1.11–1.35) in the adjusted model when TG levels were <5.6 mmol/L (**Table 3**). Similarly, when TC levels were <6.5 mmol/L, a per-unit increase in RC levels was associated with a 1.42 times increased risk of developing GDM (RR = 1.42; 95% CI = 1.27–1.84). In cases where LDL-C and HDL-C levels were within normal ranges, the RRs for GDM after adjusting for potential confounders were RR = 1.21 (99% CI = 1.10–1.32) and RR = 1.26 (95% CI = 1.17–1.35), respectively.

**Table 3:** Association between RC and GDM when TC, TG, LDL-C and HDL-C are at normal levels

	Model 1*		Model 2†	
**Items**	**RR (95% CI)**	***P-*value**	**RR (95% CI)**	***P-*value**
TG<5.6 mmol/L	1.18 (1.07–1.31)	0.001	1.22 (1.11–1.35)	<0.001
TC<6.5 mmol/L	1.39 (1.24–1.56)	0.001	1.42 (1.27–1.84)	<0.001
LDL-C<3.4 mmol/L	120 (1.10–1.31)	<0.001	1.21 (1.10–1.32)	<0.001
HDL-C≥1.0 mmol/L	1.25 (1.16–1.34)	<0.001	1.26 (1.17–1.35)	<0.001

CI – confidence interval, HDL-C – high-density lipoprotein cholesterol, LDL-C – low-density lipoprotein cholesterol, RC – remnant cholesterol, RR – relative risk, TC – total cholesterol, TG – triglyceride

*Model 1 crude.

†Model 2 adjusted for maternal age, educational level, occupation, parity, gravidity, pre-pregnancy BMI, and hypertensive disorders of pregnancy.

### Stratification analysis on the association between RC level and risk of GDM

The results of subgroup analyses, stratified by maternal advanced age, occupation, gravidity, parity, pre-pregnancy BMI, and HDP, were consistent with the main results. There were no significant interactions observed between any of the subgroup factors and the risk of GDM (*P* > 0.05). However, it is worth noting that the association between RC level and the risk of GDM appeared to be stronger in pregnant women who were not employed, had high gravidity, were multiparous, were overweight or obese, and had HDP (Table S1 in the **Online Supplementary Document**).

### Mediating role of inflammation-related indicators

Mediation analysis revealed that leukocytes mediated 4.8% of the relationship between RC levels and the risk of GDM (*P* < 0.001). Furthermore, neutrophils were also found to partially mediate the association between RC levels and the risk of GDM, accounting for 6.6% of the mediation (*P* < 0.001). However, no mediating effect was observed when considering lymphocytes and monocytes as the mediating variables (Table S2 in the **Online Supplementary Document**).

### Sensitivity analysis

We conducted several sensitivity analyses, including the introduction of RC level tertile as a categorical variable into the logistic regression model. The results showed that the overall trend of RC level on the risk of GDM remained consistent with the results when RC was classified as quartiles (Table S3 in the **Online Supplementary Document**). Furthermore, we performed another sensitivity analysis among pregnant women with a BMI < 24.0 kg/m^2^, among women without hyperthyroidism, hypothyroidism, and women with the co-occurrence of GDM and HDP (Table S4 in the **Online Supplementary Document**). Specifically, when excluding pregnant women with thyroid dysfunction or the co-occurrence of GDM and HDP, the RRs of GDM in the high quartile of RC level were still significant.

## DISCUSSION

Based on a prospective birth cohort study of 13 446 pregnant women, we observed a linear upward trend in the risk of GDM with increasing serum RC levels during the first trimester of pregnancy. The incidence of GDM rose across the quartiles of RC levels, reaching 25.7% in the highest quartile. Compared to the lowest RC quartile, higher quartiles were associated with increased risks of GDM, with RR = 1.20 (95% CI = 1.07–1.36), RR = 1.24 (95% CI = 1.11–1.40), and RR = 1.48 (95% CI = 1.32–1.67) for Q2, Q3, and Q4, respectively, in the crude model. This positive association persisted even after adjusting for TC, LDL-C, and HDL-C levels. A one-unit and one-SD increase in RC levels was associated with a 124% and 114% increased risk of GDM after adjusting for potential confounding factors. Furthermore, this association was still significant across groups stratified by maternal advanced age, occupation, gravidity, parity, pre-pregnancy BMI, and HDP. Sensitivity analysis supported the stability of these positive associations. Additionally, our results indicated that inflammation-related indicators, such as leukocytes and neutrophils, partially mediated these associations.

The results of the current study are consistent with the three recent studies showing that RC levels are associated with an increased risk of GDM. A prospective cohort study of 2528 pregnant women found that elevated RC levels at 16–17 weeks of gestation were associated with an increased risk of GDM and were identified as an independent factor [23]. Another secondary analysis using data from a prospective cohort study in Korea demonstrated that high RC levels at 12–14 weeks of gestation were linked with a high risk of developing GDM. It was also found that pregnant women with an RC level ≥24.30mg/dL were at a higher risk of GDM [22]. Similar conclusions were drawn from a prospective birth cohort study conducted between 2018–21, where RC levels at 6–13 (+ 6) gestational weeks increased the likelihood of developing GDM [27]. These findings suggest that more attention should be paid to RC levels during early pregnancy to identify the risk of GDM.

Elevated lipid levels during pregnancy are considered normal physiologic changes, and higher lipid levels are associated with an increased risk of GDM. Pregnant women with GDM have increased levels of TC, TG, and LDL-C and decreased levels of HDL-C compared to pregnant women without gestational diabetes [32]. Additionally, the most commonly used cholesterol markers in clinical practice to assess lipid levels are TC, LDL-C and HDL-C. However, we found that even among those with a normal level of TC, TG, LDL-C and HDL-C, RC still poses a threat to GDM. Therefore, when the traditional lipid levels are normal, we still need to be concerned about residual cholesterol. RC, as an untraditional lipid parameter, has been proven to be associated with type two diabetes mellitus and glucose metabolism and is better than traditional lipid parameters for predicting hyperglycaemia [14,33–36]. Several possible mechanisms may explain the associations between RC levels and GDM. RC is a cholesterol consisting of very low-density lipoproteins, intermediate-density lipoproteins, and chylomicrons, which can directly contribute to insulin resistance (IR) [18]. Ohnishi et al. found that fasting RC was strongly associated with IR among residents in a rural community [17]. Another study investigated the relationship between postprandial remnant-like particle metabolism and IR, indicating that postprandial RC was closely related to IR, regardless of age, BMI, and other lipid profiles [21]. Although the mechanisms underlying the association between RC levels and IR are not yet fully understood, RC may contribute to IR abnormalities by increasing levels of inflammation [12,37,38], as supported by our study. We also observed that leukocytes and neutrophils played a significant mediating role in the relationship between RC levels and the risk of GDM. Constituents such as very low-density lipoprotein cholesterol promote inflammatory responses by stimulating interleukin-1β [39]. Evidence has found that inflammatory cytokines may damage the insulin secretion and sensitivity, and further cause IR and *β*-cell dysfunction [40]. Similarly, we speculate that neutrophils may also be RC-promoted inflammatory responses, causing GDM. Another possible explanation may be the toxicity of cholesterol. RC particles are larger and contain more cholesterol than LDL-C particles, which may directly damage pancreatic *β*-cells, leading to insulin secretion and even apoptosis of *β*-cells [41–43].

Higher lipid levels were often observed among women with overweight and obesity, especially during pregnancy [23]. Additionally, the stratified interaction analysis revealed the risk of GDM associated with RC was higher among pregnant women with no occupation, high gravidity, multiparous, overweight and with HDP, which may be partly explained by subnormal RC levels among this study population. Women with overweight and obesity had the highest risk of GDM, suggesting that the elevated pre-pregnancy BMI is associated with an increased risk of GDM, with the risks being more pronounced among women who were overweight and obese prior to pregnancy. Besides, pregnant women with high gravidity, multiparous status, and HDP may experience more varied physiological changes, such as an increased inflammation response, which may strengthen the association between RC and the risk of GDM. Further experimental studies may reveal a deeper mechanism. In addition, based on the mediating role of these inflammatory markers in the prevalence of GDM, future trials related to anti-inflammatory or lipid-lowering in pregnancy to intervene in the development of GDM should be further explored. Higher physical activity is inversely associated with GDM risk, while unemployed women have lower physical activity, which may explain the stronger associations among unemployed women [26]. Further studies are needed to explore the effects of RC and GDM in these populations.

We revealed that elevated RC levels were independently associated with an increased risk of GDM. It is recommended that in addition to traditional blood markers such as TG, TC and others, RC and other lipid markers should also be used as additional biomarkers to identify GDM risk, even though TC, TG, LDL-C, and HDL-C were within normal levels. As the RC level is readily available from hospital-based lipid testing at no additional cost, it makes primary prevention more practicable. More attention should be paid to monitoring and managing RC in the future. Adherence to a balanced diet and appropriate physical activity is recommended as these measures can improve insulin sensitivity and reduce lipid levels, especially in individuals with a high pre-pregnancy BMI, a high number of pregnancies, postpartum mothers, and individuals with comorbid HDP.

Reducing maternal mortality and promoting maternal health are complex public health tasks [44]. Currently, maternal mortality is stagnant globally. The majority of maternal deaths are preventable, and direct obstetric complications (*e.g.* postpartum haemorrhage, preeclampsia, and infections) continue to be the leading biomedical cause of maternal deaths [45]. China has established a comprehensive health service system for mothers and children, combining disease prevention and clinical treatment functions to promote the health of mothers and children and, in particular, to reduce maternal mortality [46]. We aimed to identify modifiable biomarkers that can improve maternal health, consistent with the Sustainable Development Goals (goal three: good health and well-being), and are essential for improving maternal health and well-being and reducing maternal mortality.

The data used for this study are from a large prospective birth cohort study, providing initial insights into studying the relationship between RC and GDM risk. This study is the first to investigate the role of inflammation-related indicators in mediating the association between RC levels and the risk of GDM among pregnant women. To ensure the robustness of the results, several sensitivity analyses were conducted, including categorising RC levels into tertiles, excluding pregnant women with BMI≥24.0 kg/m^2^, hyperthyroidism, hypothyroidism and women with concurrent GDM and HDP, as well as conducting subgroup analyses. However, several limitations must be considered. First, as the study population was sampled from a single hospital, the generalisability of the results to other populations is limited. Further cohort studies in different dietary habits, health care systems, and socioeconomic factors populations are needed to validate our findings. Second, although we adjusted for some potential confounders influencing the relationship between RC and GDM, other factors associated with insulin sensitivity were not included due to incomplete data. More prospective studies are needed in the future to determine the association between RC and GDM, taking into account dietary factors. Third, other inflammatory markers, such as C-reactive protein, were collected for mediation analysis. Finally, as food intake can significantly influence RC levels, the lack of dietary data may be susceptible to bias. Future studies that include detailed dietary assessments would strengthen the study.

## CONCLUSIONS

In pregnant women, serum RC level in early pregnancy is positively associated with risk of GDM and this positive association still existed when TC, LDL-C, HDL-C are at normal levels. Leukocytes and neutrophils partially mediated these associations. More attention should be paid to monitoring and management of RC beyond TG, TC, HDL-C and LDL-C, especially those with high RC in early pregnancy.

## Additional material


Online Supplementary Document
