# Supplementary material for: The mediating role of inflammation-related indicators in the association of remnant cholesterol with gestational diabetes mellitus
Source: J Glob Health. 2025 Jun 20;15:04172. doi: 10.7189/jogh.15.04172 (PMC12178596; doi:10.7189/jogh.15.04172)
Supplement: Online Supplementary Document [file jogh-15-04172-s001.pdf]

Supplement to: Lin L, Lin J, Yan J, Wang X. The mediating role of inflammation-related indicators in the association of remnant cholesterol with gestational diabetes mellitus. J Glob Health. 2025;15:04172.

Table S1. Stratification analysis on the association between RC level with risk of GDM.

**Subgroup**	**GDM(%)**	**RC Quartile**				P_{trend}	$P_{interaction}$
		Quartile1	Quartile2 RR(95%CI)	Quartile3 RR(95%CI)	Quartile4 RR(95%CI)		
Advanced age							0.392
<35	609(35.1)	Ref(1.00)	1.37 (1.02~1.83)	1.45 (1.09~1.93)	1.5 (1.13~2.01)	<0.001	
≥35	2385 (20.4)	Ref(1.00)	1.24 (1.09~1.42)	1.24 (1.08~1.41)	1.53 (1.34~1.75)	0.084	
Occupation							0.24
Yes	1984 (22)	Ref(1.00)	1.18 (1.02~1.37)	1.24 (1.07~1.44)	1.45 (1.25~1.67)	<0.001	
No	1010 (22.9)	Ref(1.00)	1.28 (1.03~1.59)	1.5 (1.22~1.84)	1.69 (1.37~2.08)	<0.001	
Gravidity							0.199
1	1218 (19.3)	Ref(1.00)	1.28 (1.07~1.53)	1.28 (1.06~1.54)	1.3 (1.08~1.57)	0.01	
2	915 (23.3)	Ref(1.00)	1.24 (0.98~1.55)	1.3 (1.03~1.64)	1.73 (1.39~2.15)	<0.001	

≥3	861 (26.8)	Ref(1.00)	1.26 (1.01-1.60)	1.28 (1.01~1.62)	1.65 (1.32~2.07)	<0.001	
Parity							0.051
Primiparous	1802 (20.5)	Ref(1.00)	1.27 (1.09~1.48)	1.3 (1.12~1.51)	1.38 (1.19~1.61)	<0.001	
Multiparous	1192 (25.7)	Ref(1.00)	1.26 (1.03~1.56)	1.27 (1.03~1.57)	1.76 (1.44~2.14)	<0.001	
Pre-pregnancy BMI							0.623
Underweight	536 (23)	Ref(1.00)	1.11 (0.84~1.48)	1.15 (0.86~1.53)	1.38 (1.04~1.83)	0.039	
Normal weight	2125 (22.1)	Ref(1.00)	1.29 (1.11~1.49)	1.36 (1.18~1.57)	1.53 (1.33~1.77)	<0.001	
Overweight and obese	333 (22)	Ref(1.00)	1.03 (0.71~1.5)	1.29 (0.89~1.88)	1.66 (1.17~2.36)	0.002	
HDP							0.314
Yes	277 (32.2)	Ref(1.00)	1.22 (0.78~1.93)	1.61 (1.06~2.45)	2.0 (1.31~3.04)	0.007	
No	2717 (21.6)	Ref(1.00)	1.25 (1.1~1.42)	1.25 (1.1~1.42)	1.48 (1.31~1.68)	<0.001	

Abbreviations:BMI–body mass index, CI:confidence intervals, GDM–gestational diabetes mellitus, HDP–hypertensive disorders of pregnancy, RC–remnant cholesterol,RR–Relative risk .
Adjusted for maternal age, educational level ,Occupation, parity , gravidity, pre-pregnancy body mass index, hypertensive disorders of pregnancy.

Table S2 Mediation analyses of association between maternal RC and GDM.

Variable	Total effect(95%CI)	Natural direct effect(95%CI)	Natural indirect effect(95%CI)	Mediated proportion,%
lymphocytes	0.03789(0.02533~0.05045)***	0.03857(0.02598~0.05115)***	-0.00068(-0.00157~-0.000213)	1.7870
leukocytes	0.04159(0.03433~0.06245)***	0.03974(0.02719~0.05229)***	0.00185(0.00274~0.00096)***	4.8884***
monocytes	0.03789(0.02533~0.05045)	0.04133(0.02878~0.05389)***	-0.00345(-0.00465~-0.00224)	-9.0931***
neutrophils	0.04289(0.03033~0.05545)***	0.04039(0.02783~0.05295)***	0.00250(0.00153~0.00347)***	6.5996***

Abbreviations:CI–confidence intervals, GDM–gestational diabetes mellitus,RC–remnant cholesterol.
***: P<0.001

Table S3 Associations between maternal RC levels in the frst trimester and the risk of GDM when RC level was classified as tertiles.

Variable	GDM(%)	Model1		Model2	
		Crude RR(95%CI)	*P* value	Adjusted RR(95%CI)	*P* value
RC tertile1	921 (20.5)				
RC tertile2	946 (21.5)	1.06 (0.96~1.18)	0.244	1.1 (0.99~1.22)	0.071
RC tertile3	1127 (24.8)	1.28 (1.16~1.41)	<0.001	1.31 (1.18~1.45)	<0.001
P for trend			<0.001	1.14 (1.09~1.2)	<0.001

Abbreviations:CI–confidence intervals, GDM–gestational diabetes mellitus, RC–remnant cholesterol, RR–Relative risk, .
Model 1: crude;Model 2：adjusted for maternal age, educational level ,Occipation, parity , gravidity, pre-pregnancy body mass index, hypertensive disorders of pregnancy.

Table S4 Associations between maternal RC levels in the frst trimester and the risk of GDM after excluding some study population.

	RC level RR(95%CI)	Quartile1	Quartile2 RR(95%CI)	Quartile3 RR(95%CI)	Quartile4 RR(95%CI)
Excluding women with pregnancy overweight or obese	1.25(1.16~1.36)	Ref(1.00)	1.31(1.15~1.49）	1.24（1.09~1.42）	1.49（1.32~1.69）
Excluding women with hypothyroidism	1.24（1.15~1.33）	Ref(1.00)	1.24（1.10~1.41）	1.29（1.14~1.46）	1.53（1.35~1.72）
Excluding women with hyperthyroidism	1.23（1.15~1.32）	Ref(1.00)	1.23（1.08~1.39）	1.27（1.13~1.44）	1.51（1.34~1.70）
Excluding women with co-occurrence of GDM and HDP	1.22(1.14~1.31)	Ref(1.00)	1.25(1.10~1.42)	1.26(1.11~1.42)	1.48(1.31~1.68)

Abbreviations:CI:confidence intervals, GDM–gestational diabetes mellitus,RC–remnant cholesterol, RR–Relative risk, .

Adjusted for maternal age, educational level ,Occipation, parity , gravidity, pre-pregnancy body mass index, hypertensive disorders of pregnancy.
